# Quasi-static stop band with flexural metamaterial having zero rotational stiffness

**DOI:** 10.1038/srep33410

**Published:** 2016-09-21

**Authors:** Joo Hwan Oh, Badreddine Assouar

**Affiliations:** 1 University of Lorraine, Institut Jean Lamour, Boulevard des Aiguillettes, BP: 70239, Vandoeuvre-lès-Nancy, 54506, France; 2 CNRS, Institut Jean Lamour, Vandoeuvre-lès-Nancy, 54506, France; 3 Institute of Advanced Machine and Design, Seoul National University, 599 Gwanak-ro, Gwanak-gu, Seoul, 151-744, Korea

## Abstract

Metamaterials realizing stop bands have attracted much attentions recently since they can break-through the well-known mass law. However, achieving the stop band at extremely low frequency has been still a big challenge in the fields of elastic metamaterials. In this paper, we propose a new metamaterial based on the idea of the zero rotational stiffness, to achieve extremely low frequency stop band for flexural elastic waves. Unlike the previous ways to achieve the stop band, we found that the zero rotational stiffness can provide a broad stop band at extremely low frequency, which starts from even almost zero frequency. To achieve the zero rotational stiffness, we propose a new elastic metamaterial consisting of blocks and links with the hinge connection. Analytic developments as well as numerical simulations evidence that this new metamaterial can exhibit extremely low and broad stop band, even at the quasi-static ranges. In addition, the metamaterial is shown to exhibit the negative group velocity at extremely low frequency ranges, as well as the quasi-static stop band, if it is properly designed.

Recently, researches on the artificial sub-wavelength acoustic or elastic structures, called acoustic/elastic metamaterials, have extended our knowledge beyond the current limit. By utilizing the intrinsic dynamic motions inside their unit cell structures, metamaterials have shown various interesting wave phenomena, such as the negative refraction or even the wave cloaking, which were impossible to be realized previously. Above all, the metamaterial can be designed to have the stop band that can break-through the well-known mass-law, which is very important characteristic in various vibration/acoustic devices. In fact, the break-through of the mass-law has been already shown in the phononic crystals^1^,^2^, which utilize the Bragg scattering to achieve a stop band originated from the crystal’s periodicity^3^,^4^. However, there has been a limitation that the stop bands in phononic crystals generally exist at too high frequencies to be applied in vibration or acoustic devices.

On the other hand, recent researches on the resonance-based metamaterials have realized a stop band at relatively low frequencies where the wavelength is much larger than the size of the unit cell. From the first realization of the resonance-based acoustic metamaterial by Ping Sheng *et al*.^5^, there have been a number of researches on the resonance-based metamaterials. For the in-plane elastic waves, Huang *et al*.^6^ developed an analytic theory to explain the formation of the negative density with the resonance-based metamaterials. Zhu *et al*.^7^ realized the anisotropic elastic metamaterial having different wave stop band for different directions. Hou *et al*.^8^ proposed the idea of the tunable elastic metamaterial having negative stiffness. Zhu *et al*.^9^,^10^ realized the chiral elastic metamaterials having broad stop band and double negativity, respectively. Romero-Garcia *et al*.^11^ realized the resonance-based acoustic metamaterial having various stop band. Recently, Oh *et al*.^12^,^13^ realized the elastic metamaterial whose negative density and stiffness can be independently realized and adjoin each other, respectively. For the out-of-plane elastic waves (flexural elastic waves), Oudich *et al*.^14^ realized the locally resonant metamaterial with the stubbed plates. Williams *et al*.^15^ developed the multiresonant metamaterials for the flexural waves. Assouar *et al*.^16^ proposed the double-sides stubbed plates for broad stop band. The latter extended the idea of the double-sides stubbed plates to the hybrid plates having both cylinders and holes to further enlarge the stop band^17^. In addition, there have been several researches to achieve the extremely low frequency stop band without the internal resonance phenomena. Li *et al*.^18^ utilized the concept of the acoustic coiling-up space to achieve perfect absorption and zero transmission at very low frequency without the internal resonance metamaterial. Oh *et al*.^19^ used the idea of the corrugated elastic structure to obtain broad stop band at very low frequency ranges, and extended the idea to achieve the far field sub-wavelength imaging lens.

Despite of the previous researches on the low frequency stop band metamaterials, challenge still remains on achieving much lower frequency stop band, especially the stop band covering almost quasi-static frequency ranges. In fact, there have been only few researches on the metamaterials that allow the quasi-static stop band. In acoustics, Lee *et al*.^20^,^21^ proposed and realized the acoustic metamaterial having negative density and bulk modulus at the quasi-static frequencies, respectively. However, due to the physical difference between acoustics (based on the vector physics) and elasticity (based on the tensor physics), the acoustic metamaterials cannot be directly used to achieve the elastic quasi-static stop band. In elasticity, Yao *et al*.^22^ and Yu *et al*.^23^ utilized the idea of the elastic foundation to realize the elastic quasi-static stop band. Nevertheless, the elastic metamaterials require the connection with the external fixed boundary to achieve the quasi-static wave stop band. For the best of the author’s knowledge, there has been no research to obtain the elastic metamaterial with the quasi-static stop band by its intrinsic dynamic characteristics only.

In this research, we proposed a new metamaterial to obtain the elastic quasi-static stop band for out-of-plane flexural elastic waves. The main idea in this research is called ‘the zero rotational stiffness,’ which is completely different from the previous metamaterials based on the internal resonance, the coiling up space or the Bragg scattering. Here, from the classical Timoshenko beam theory, we found that the broad quasi-static stop band metamaterial can be achieved if the metamaterial has a zero rotational stiffness. Based on this idea, we newly designed a new elastic metamaterial, consisting of the hinge connection based structure with blocks and links, to achieve the zero rotational stiffness. The analytic theory and the numerical simulations for the metamaterial showed that the metamaterial has broad quasi-static stop band from almost zero frequency. Moreover, with proper design, we found that the metamaterial can be tuned to exhibit negative group velocities in addition to the quasi-static stop band. Since extremely low frequency stop band can be obtained with this research, the findings in this paper are expected to be further applied in various vibration and flexural wave devices.

## Results

### Background theory: mass-spring system for general flexural waves

**Figure 1:**
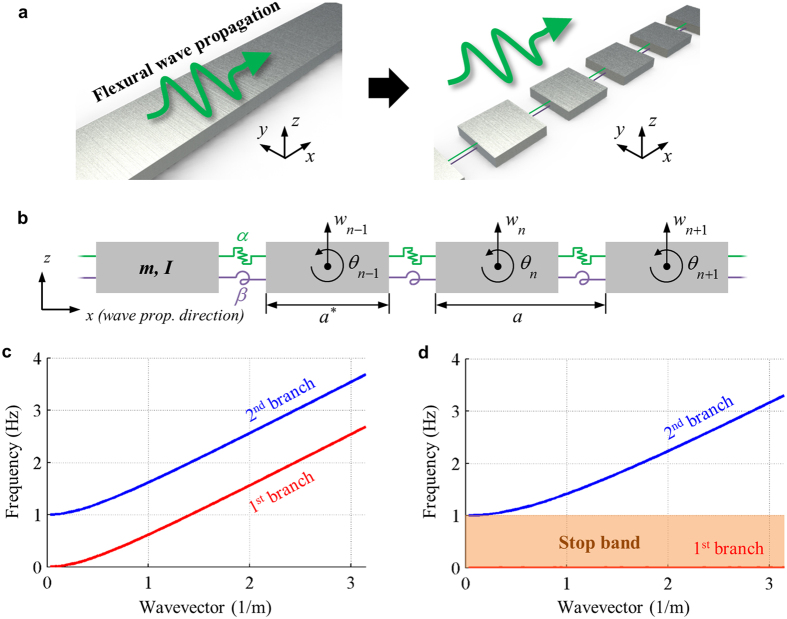
(**a**) Conversion of general beam under flexural wave motion to the mass-spring system, (**b**) general mass-spring system for flexural wave, (**c**) typical wave dispersion curve of flexural wave in general beam, (**d**) wave dispersion curve of flexural wave with the zero rotational stiffness.

Before showing the main idea of the zero rotational stiffness, it is worth to review the periodic mass-spring system for the general flexural wave. Consider a flexural wave propagating through a slender beam as in Fig. 1(a). To analyze the wave dispersion relation of the flexural wave, this slender beam can be converted to the equivalent mass-spring system shown in Fig. 1(b). It should be emphasized that each mass in Fig. 1(b) are connected with two springs. In acoustics or in-plane elastic waves, only one spring connecting each mass is considered in the equivalent mass-spring system. However, as well known from the classical Timoshenko beam theory, the flexural wave is governed by two types of motions, the material’s vertical movement *w* and the rotation *θ*^24^. Accordingly, in the equivalent mass-spring system of the flexural waves, both springs, the vertical shear spring *α* (which represents the shear stiffness of the beam) and the rotational spring *β* (which represents the bending stiffness of the beam)^25^, should be considered between each mass to properly model the flexural waves. Also, both the mass *m* and the momentum of inertia *I* are considered for each mass. Following the detailed procedures shown in the Supplementary Material, the equation of motion of the equivalent mass-spring system can be written as
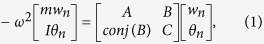

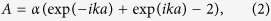

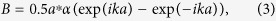

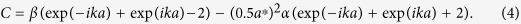
Here, *ω* and *k* are the angular frequency and the wavevector, respectively.

In homogeneous continuous beam structure, Equations (1, 2, 3, 4) can be further reduced by using the fact that *a* = *a* ≪ 1. In this case, exp(*ika*) + exp(−*ika*)~2 − (*ka*)^2^ and exp(*ika*) − exp(−*ika*)~2*ika* can be assumed. As a result, the wave dispersion curve can be obtained as (see the Supplementary Material for the detailed procedures)
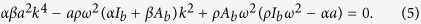
As can be seen in Equation (5), unlike those in acoustics or in-plane elastic waves, the wave dispersion equation of the flexural wave has a fourth order equation. Figure 1(c) shows the plot of the dispersion curves shown in Equation (5). Here, *A*_*b*_, *I*_*b*_, *ρ*, *a*, *α* and *β* are considered to be 1. As can be seen here, there are two wave dispersion curves for general flexural waves. The first dispersion branch starts from the origin, i.e., the zero frequency. On the other hand, the second dispersion branch starts to appear above the certain cutoff frequency.

### Main idea of the zero rotational stiffness

Based on the wave dispersion relation of general flexural wave, let us clarify our idea. The main idea in this work, the zero rotational stiffness, is to make the rotational spring *β* to be zero. From Equation (5), it can be clearly seen that if the rotational spring *β* is zero, the 4^th^ order term (the *k*^4^ term) in the wave dispersion equation becomes zero and the wave dispersion equation is reduced to the second order equation as
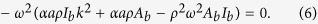
Accordingly, the wave dispersion equations for the zero rotational stiffness are
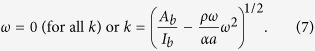
Figure 1(d) shows the wave dispersion equation with *β* = 0 while the other constants are same as in Fig. 1(c). From Fig. 1(d), one can clearly see that if *β* = 0, the first branch becomes totally flat while the second branch is almost unaltered. Thus, one can obtain the quasi-static stop band below the cutoff frequency of the second branch.

As explained, the idea of the zero rotational stiffness is a totally different mechanism compared to the Bragg scattering and the local resonance phenomena that have been utilized to achieve the stop band previously. The previous ideas of the Bragg scattering and the local resonance phenomena are to create a stop band at a frequency range which was pass band naturally. However, in this research, we don’t create a stop band; we make the first dispersion branch to be a flat branch so that we can utilize the existing cutoff frequency of the second branch as a stop band. Also, the idea of the zero rotational stiffness is valid for the flexural wave only. In acoustic or in-plane elastic waves, if the bulk modulus or the corresponding stiffness is zero, the stiffness term becomes zero for the whole frequency and the wave motion, such as the wave dispersion equation, cannot be defined. However, because the flexural wave is governed by both the vertical shear spring and the rotational spring, although the rotational spring is zero, each component in the stiffness matrix is not zero unless the vertical shear spring is zero too. Thus, the wave dispersion equation can still be defined although *β* = 0.

### Metamaterial design to achieve the zero rotational stiffness

**Figure 2:**
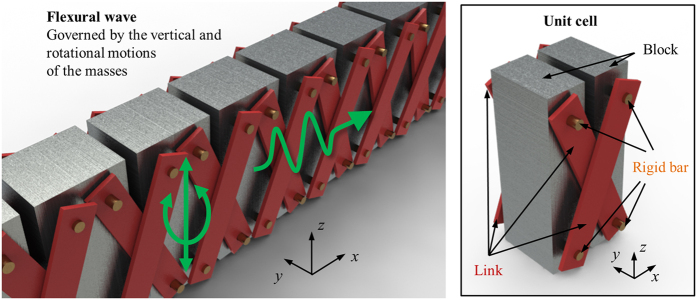
The proposed unit cell structure having zero rotational stiffness.

There can be various metamaterial designs to achieve the zero rotational stiffness. Here, among the various ways, we consider a hinge connection based structure to achieve the zero rotational stiffness. The metamaterial design consisting of the hinge connection is shown in Fig. 2. In the current metamaterial design, the most important part is the rigid cylinder that connects the blocks and the links. The rigid cylinder connecting the blocks and the links makes two structures move together along *x*, *y* and *z* directions. However, the rigid cylinder does not provide any constraints for their rotation with respect to the cylinder. Thus, if any moment is induced, the links and the blocks do not deform but rotate independently. Therefore, no moment can be transferred between the blocks and the links, i.e., the structure has the zero rotational stiffness.

### Analytic modeling of the metamaterial

**Figure 3:**
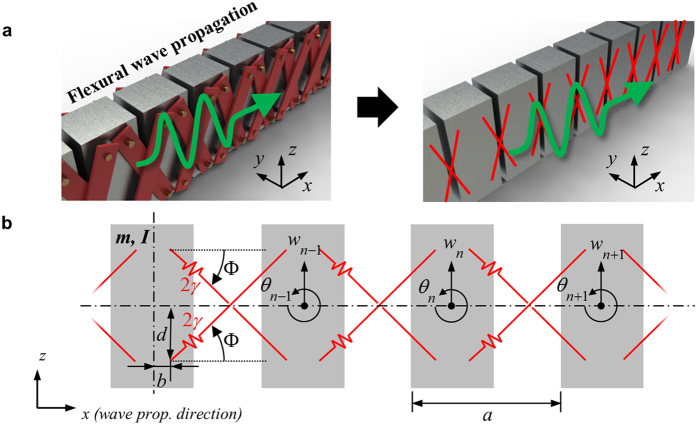
(**a**) Conversion of the proposed metamaterial to the mass-spring system, (**b**) the equivalent mass-spring system of the proposed metamaterial.

Based on the proposed metamaterial, let us focus on the analytic investigation of the metamaterial first. To carry out this analytic investigation, the metamaterial shown in Fig. 2 is replaced by the equivalent mass-spring system as in Fig. 3(a). Here, we will focus on the linear case only when the motion of the blocks is small and thus the change of the angle of the links Φ is ignorable during the motion. In the equivalent mass-spring system in Fig. 3(b), it is assumed that the dominant deformation takes place at the links while the blocks exhibit almost zero deformation. This assumption is usually valid since the links generally have a low stiffness than the blocks. Also, the rotational springs are not used to describe the links since the links are connected with the blocks by the hinge connection and thus no moment can be transferred from the links to the blocks. As a result, the links are modeled as the longitudinal spring with the spring coefficient *γ*. From the detailed analytic procedures shown in the Supplementary Material, the wave dispersion equation can be derived as
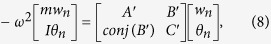

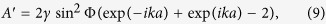

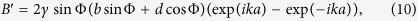

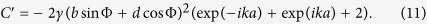


Comparing Equation (8) with the general wave dispersion equation in Equation (1), one can find that two equations are in fact identical to each other with the following equivalences;
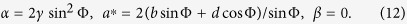
Here, one can clearly see that the metamaterial is equivalent to the general mass-spring system with *β* = 0, i.e., the proposed unit cell structure has indeed the zero rotational stiffness. Also, it can be seen that the angle of the links Φ should be non-zero value; otherwise, the stiffness matrix in Equation (8) becomes zero for all frequencies and the wave phenomena inside the metamaterial cannot be defined.

### Numerical methodology for the metamaterial

**Figure 4:**
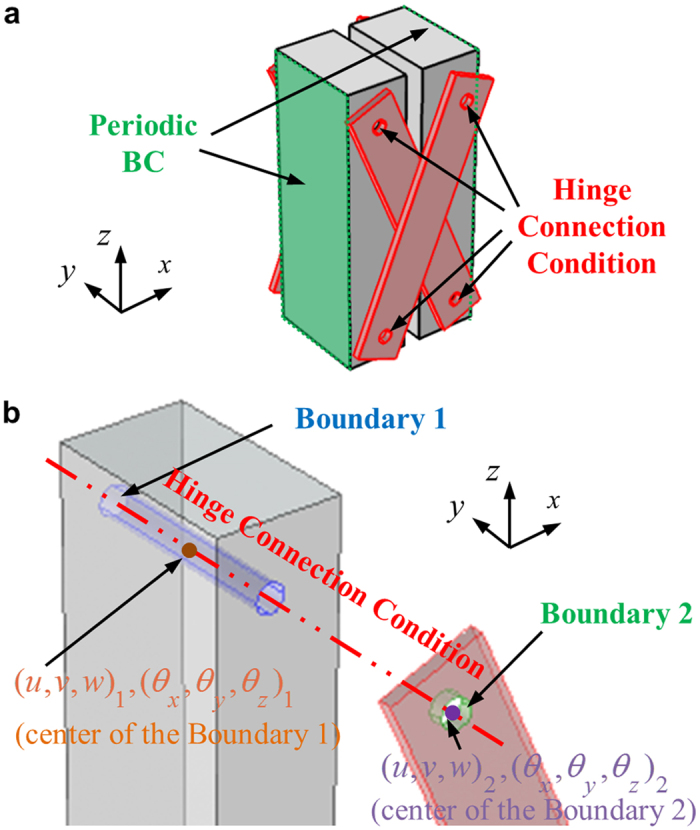
(**a**) Continuum model of the proposed metamaterial’s unit cell, (**b**) the hinge connection condition imposed in the current numerical simulation.

Now, the numerical methodology to evaluate the wave dispersion curves of the continuum unit cell structure is focused. Figure 4(a) shows the actual continuum model used in the current numerical investigation. Here, aluminum (Young’s modulus = 70 GPa, Poisson’s ratio = 0.33 and density = 2700 kg/m^3^) is considered for the blocks and the polytetrafluoroethylene (PTFE, Young’s modulus = 0.5 GPa, Poisson’s ratio = 0.49 and density = 2000 kg/m^3^) is considered for the links. As used for general metamaterials, the Floquet-Bloch boundary condition is applied at the sides of the unit cell in Fig. 4(a) and the eigenvalue problem is solved for various wavevectors *k* inside the 1^st^ irreducible Brillouin zone^26^. In the simulation, the structural module (linear analysis) of COMSOL 4.3b is used.

In the current numerical simulation, a special attention should be paid to properly model the hinge connection condition. To explain how the hinge connection is modeled, let us consider the hinge connection between only one block and link, as in Fig. 4(b). Here, Boundary 1 indicates the contact boundary of the block with the rigid cylinder, and Boundary 2, that of the link with the rigid cylinder. To introduce the hinge connection condition, all displacements of the nodes at the Boundary 1 are expressed by the displacements and the rotations of the point located at the center of the Boundary 1, respectively. For instance, the displacement **U** = (*u*, *v*, *w*)_*n*_ of any node at the Boundary 1 can be expressed as
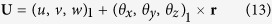
where (*u*, *v*, *w*)_1_ and (*θ*_*x*_, *θ*_*y*_, *θ*_*z*_)_1_ are the displacements and the rotations of the center of the Boundary 1, as in Fig. 4(b). Also, **r** is the vector which connects from the center to the node in the Boundary 1. In the same manner, all the displacements of the nodes at the Boundary 2 are expressed by (*u*, *v*, *w*)_2_ and (*θ*_*x*_, *θ*_*y*_, *θ*_*z*_)_2_, which are defined at the center of the Boundary 2. After that, the following constraints are imposed between two center points of the Boundary 1 and 2 to achieve the hinge connection condition;
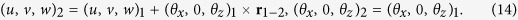
Here, **r**_1−2_ is the vector which connects from the center of the Boundary 1 to the center of the Boundary 2. As can be seen in Equation (14), the displacements and the rotations of the center of the Boundary 1 are set to be identical to those of the center of the Boundary 2, except the rotation with respect to the *y* axis, (*θ*_*y*_)_1_ and (*θ*_*y*_)_2_. Therefore, the block and the link can freely rotate independently with respect to the *y* axis, i.e., (*θ*_*y*_)_1_ and (*θ*_*y*_)_2_ are perfectly independent to each other. By this way, one can model the hinge connection condition numerically.

### Validation of the analytic investigations

By using the numerical method, the validation of the analytic theory is made by comparing the wave dispersion curve obtained by the analytic mass-spring system with the numerically calculated wave dispersion curve. The detailed geometric parameter of the unit cell, and the mass and spring coefficients used for the analytic calculation, are given in the Supplementary Material.

**Figure 5:**
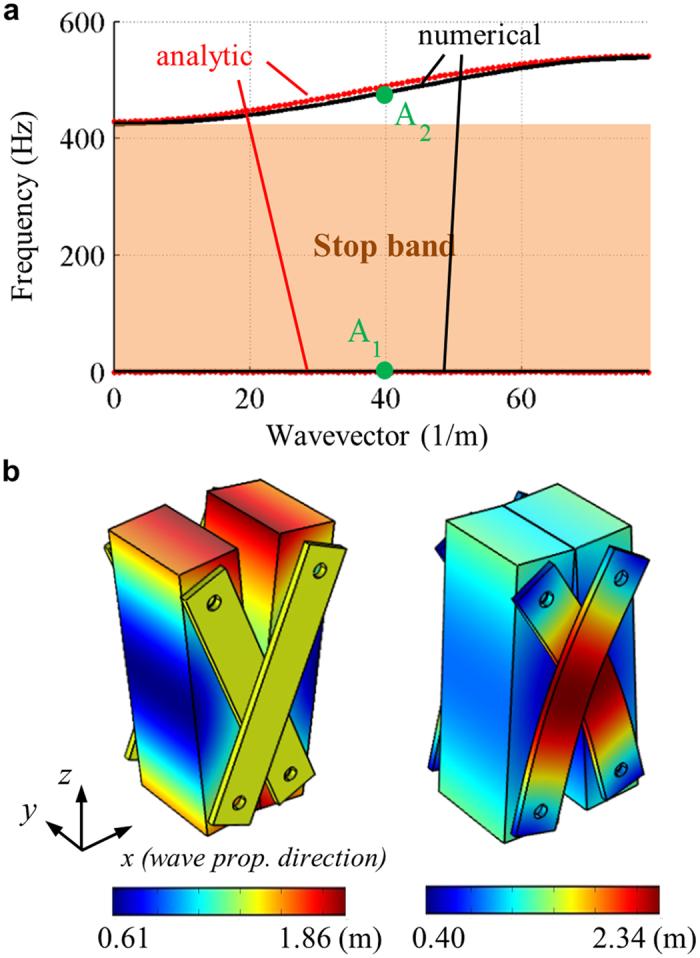
(**a**) Plot of the analytically and numerically calculated wave dispersion curves, (**b**) the mode shapes of the unit cell at the point A_1_ (left) and A_2_ (right) in (**a**), respectively. Here, the color represents the total displacement.

Figure 5(a) shows the wave dispersion curves obtained by the analytic mass-spring system (Equation (8)) and the numerical simulation. Note that in Fig. 5(a), only the flexural wave mode’s wave dispersion curves are plotted among various possible elastic wave modes. Good agreements can be found between two results, validating the analytic equations derived with the equivalent mass-spring system. Also, it can be clearly seen that very broad stop band is indeed formed at extremely low frequency. The quasi-static stop band is measured to be formed from 0.006 Hz to 425 Hz. This clearly shows that the proposed elastic metamaterial has extremely low frequency stop band, owing to the zero rotational stiffness.

### Mode shape investigation of the proposed metamaterial

For the better understanding of the underlying physics, the mode shapes for each dispersion branch are studied. Figure 5(b) shows the mode shapes at the point A_1_ and A_2_ in Fig. 5(a), respectively. From the left figure in Fig. 5(b), it can be clearly seen that the links have almost same displacement, i.e., the links do not exhibit any deformation. Considering that the links act as the spring which dominates the overall stiffness of the unit cell, almost no deformation at links indicates that the mode shape involves almost zero stiffness. Thus, the frequency of the first dispersion branch should be zero, as expected from the analytic investigation. In fact, the flat dispersion branch at zero frequency indicates that the unit cell should have an instability under the zero frequency (static condition). If a metamaterial has the zero rotational stiffness, each block can freely rotate and thus the structure cannot sustain its own weight. However, in reality, the perfectly zero rotational stiffness cannot be realized due to the friction and thus the metamaterial can be stable. For more about this issue, see the Supplementary Material.

On the other hand, in the right figure in Fig. 5(b), the deformation varies in the links, showing that there is high deformation induced in the links. Therefore, the mode shape involves nonzero stiffness and the second dispersion branch should be located at nonzero frequencies, as shown in Fig. 5(a). Note that in the right figure in Fig. 5(b), one can see that the links have also some internal bending motion. This bending motion provides additional moments and shear forces, which were not considered in the analytic model. Thus, in strict meaning, the metamaterial may not provide ‘purely zero’ rotational stiffness. However, the effects of these moments and shear forces are extremely small and ignorable; due to the hinge connection condition, the moments are not transferred to the block, and the shear forces only provide very small rotation of the block. This insignificance of the internal bending motion can be supported from the good agreements between the dispersion curves shown in Fig. 5(a); although the internal bending motion is not considered, the analytic approach can precisely predict the actual wave dispersion curve of the continuum unit cell structure. This indicates that the internal bending motion is not significant in the current metamaterial, especially in the frequency range of the interest.

### Wave transmission analysis of the proposed metamaterial

**Figure 6:**
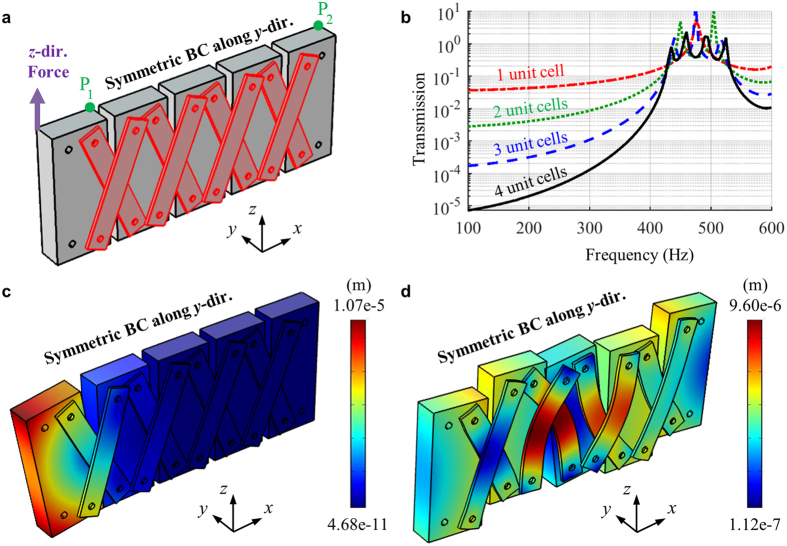
(**a**) Continuum model of the metamaterial system to measure the wave transmission, (**b**) the measured wave transmission for various number of the unit cell, total displacement plot and deformed shape of the metamaterial system at (**c**) 200 Hz and (**d**) 500 Hz.

To further validate the formation of the quasi-static stop band, the wave transmission is numerically calculated with the same program, the structural module (linear analysis) of COMSOL 4.3b. In the simulation, various metamaterial systems consisting of 1, 2, 3 and 4 unit cells, respectively, are considered. Figure 6(a) shows the continuum model consisting of 4 unit cells used for the wave transmission analysis. As in Fig. 6(a), the *z*-directional force is imposed at the left end and the *z*-directional displacements, *w*_1_ and *w*_2_, are measured at the points P_1_ and P_2_, respectively. After that, the wave transmission is evaluated as *abs*(*w*_2_/*w*_1_) for various frequencies. Although this definition of the wave transmission does not provide the exact transmission coefficient, it can clearly show the existence of the stop band. For the numerical efficiency, only the half of the structure along the *y* direction is considered and the symmetric boundary condition along the *y* direction is applied as in Fig. 6(a). Also, the frequencies over 100 Hz are considered for all simulations since there is a convergence problem at the lower frequencies due to the instability of the metamaterial.

Figure 6(b) shows the numerically calculated wave transmission for the metamaterial system consisting of various unit cells. As can be seen in the simulation results, very low wave transmissions are measured for all metamaterial system at the frequencies from 100 Hz to 425 Hz, validating the formation of the quasi-static stop band. Also, it can be seen that only few unit cells can provide very low wave transmission at the stop band frequency ranges. For instance, the wave transmission below 0.01 at 100 Hz can be achieved with only two unit cells. This suggests that the stop band in the metamaterial is not originated from the Bragg scattering. In addition, several peaks can be observed in the wave pass band from 425 Hz to 538 Hz. Considering that the number of the peaks are same as the number of the unit cells used in the metamaterial system, it can be seen that the peaks are originated from the Fabry-Perot resonance inside the metamaterial system.

To more clearly see why the stop band and the pass band are formed, the deformation characteristics of the metamaterial layer at 200 Hz (belongs to the stop band) and 500 Hz (belongs to the pass band) are shown in Fig. 6(c,d). In Fig. 6(c), it can be seen that the dominant motion of the blocks at 200 Hz is the rotation with respect to the *y* axis. Since the links do not have any stiffness for the rotation of the blocks, no wave energy can be transmitted through the metamaterial. However, at 500 Hz, the blocks exhibit not only the rotation but also the vertical movement, as can be seen in Fig. 6(d). In this case, the links exhibit internal deformation according to the vertical movement of the blocks. Thus, the wave energies can be transmitted between the neighboring blocks, making the pass band at the frequency.

In fact, from the classical Timoshenko theory, it has been well-know that the flexural wave is dominantly governed by the rotation at low frequencies, while it is governed by the coupled motion of the rotation and the vertical movement at high frequencies. Since the metamaterial has the zero rotational stiffness, it cannot transfer the wave energy at low frequencies where the wave is dominated by the rotation. However, at high frequencies, the vertical movement begins to take place and the metamaterial can transfer the wave energy due to the vertical movement. This is how the quasi-static stop band is formed with the proposed elastic metamaterial.

### Expansion to the negative group velocity

**Figure 7:**
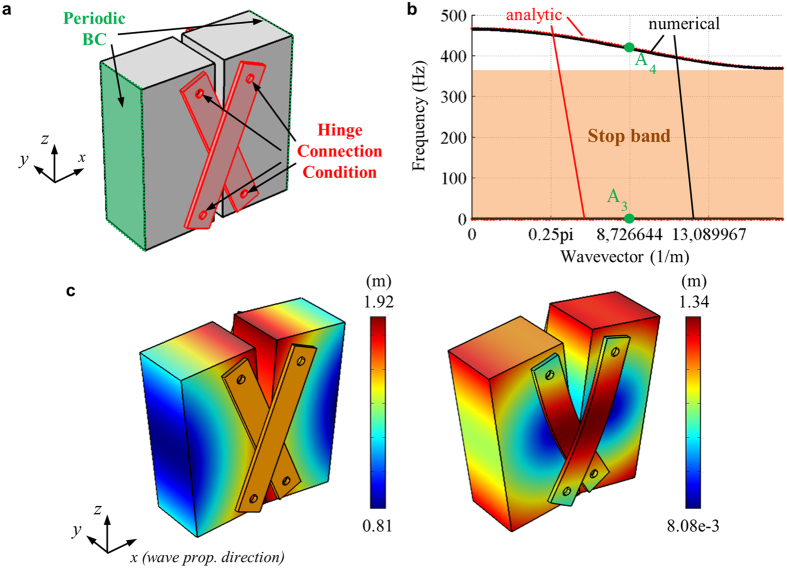
(**a**) Continuum model of the metamaterial with the negative group velocity, (**b**) plot of the analytically and numerically calculated wave dispersion curves, (**c**) the mode shapes of the unit cell at the point A_3_ (left) and A_4_ (right) in (**a**), respectively. Here, the color represents the total displacement.

In addition to the quasi-static stop band, we will show that the metamaterial can be tailored to achieve the negative group velocity. To clarify this point, Equation (8) is re-visited. From the latter, the frequencies at *k* = 0 and *k* = *π*/*a* can be analytically calculated as
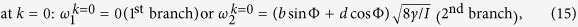

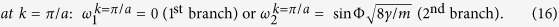
In the previously considered metamaterial, 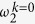 was smaller than 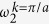 and the second dispersion branch had the positive group velocity. However, if 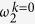 is larger than 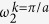, one can expect to have the negative group velocity at the second dispersion branch. Considering Equations (S29–35), one can clearly see that 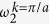 decreases as *l* increases since it will increase the total mass m. For 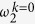, on the other hand, increasing *l* will increases *I* but it also increases *b*. In fact, in the current metamaterial, 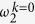 increases as *l* increases. Thus, to achieve 
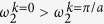
, we enlarge the length of the block as in Fig. 7(a). Figure 7(b,c) show the wave dispersion curve and the mode shapes, respectively. As can be seen in Fig. 7(b), the new metamaterial is shown to have the negative group velocity from 369 Hz to 465 Hz, in addition to the quasi-static stop band from 0 Hz to 369 Hz. This clearly supports that the proposed metamaterial can even realize negative group velocity.

**Figure 8:**
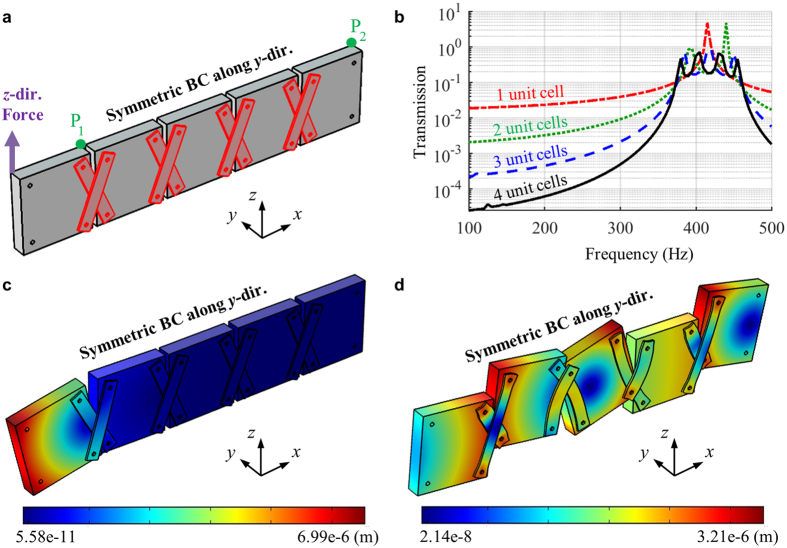
(**a**) Continuum model of the metamaterial system consisting of the metamaterial in Fig. 7(a) to measure the wave transmission, (**b**) the measured wave transmission for various number of the unit cell, total displacement plot and deformed shape of the metamaterial system at (**c**) 200 Hz and (**d**) 400 Hz.

Figure 8(a) shows the numerical model to calculate the wave transmission of the metamaterial consisting of the unit cell shown in Fig. 7(a). Here, the detailed procedures are same as that used in Fig. 6(a). The transmission plot shown in Fig. 8(b) clearly supports the wave dispersion curve in Fig. 7(b); the existence of the quasi-static stop band from 100 to 369 Hz and the pass band from 369 Hz to 465 Hz. The important point is that the formation of the negative group velocity does not affect the formation of the quasi-static stop band. In fact, from the deformation characteristics at 200 Hz (belongs to the stop band) and 400 Hz (belongs to the pass band) shown in Fig. 8(c), it can be seen that regardless of the formation of the negative group velocity, the metamaterial exhibits similar dynamic behavior as the previous metamaterial system shown in Fig. 6(c). Thus, one can achieve the quasi-static stop band and the negative group velocity simultaneously, which is favorable in various metamaterial system design. The transition from the positive to the negative group velocity by adjusting the length of the block is shown in the Supplementary Material.

## Conclusions

In this paper, we proposed a new idea of the zero rotational stiffness to achieve extremely low frequency stop band, which may cover even the quasi-static frequency range. Unlike the previous ways to achieve the stop band, such as the Bragg scattering or the internal resonance phenomena, we made the first dispersion branch of the flexural wave mode as a flat curve at the zero frequency by using the idea of the zero rotational stiffness. In this way, we found that one can achieve very broad stop band from almost zero frequency to the cutoff frequency of the second dispersion branch. As a possible way to achieve the zero rotational stiffness, we developed the new metamaterial consisting of the blocks and the links. Here, the hinge connection was introduced between the blocks and the links to achieve the zero rotational stiffness. Analytic and numerical investigations showed that the developed elastic metamaterial can exhibit very broad stop band at extremely low frequency range. In addition, further investigation showed that the metamaterial can have the negative group velocity in addition to the quasi-static stop band. Considering the importance of the extremely low frequency stop band in vibration and acoustic devices, the results in this paper is expected to open a new way in various vibration metamaterial devices. The actual realization of the metamaterial with the structure stabilization will be a subject of next researches.

## Additional Information

**How to cite this article**: Oh, J. H. and Assouar, B. Quasi-static stop band with flexural metamaterial having zero rotational stiffness. *Sci. Rep.* **6**, 33410; doi: 10.1038/srep33410 (2016).

## Supplementary Material

Supplementary Information
