# Supplementary material for: Quasi-static stop band with flexural metamaterial having zero rotational stiffness
Source: Sci Rep. 2016 Sep 21;6:33410. doi: 10.1038/srep33410 (PMC5030665; doi:10.1038/srep33410)
Supplement: Supplementary Information [file srep33410-s1.pdf]

Supplementary Materials

Quasi-static stop band with flexural metamaterial having zero rotational stiffness

Joo Hwan Oh[a,b,c] and Badreddine Assouar[a,b *]

[a] *University of Lorraine, Institut Jean Lamour, Boulevard des Aiguillettes, BP: 70239, Vandoeuvre-lès-Nancy 54506, France*

[b] *CNRS, Institut Jean Lamour, Vandoeuvre-lès-Nancy 54506, France*

[c] *Institute of Advanced Machine and Design, Seoul National University, 599 Gwanak-ro, Gwanak-gu, Seoul, 151-744, Korea*

[*] *Corresponding Author, Email: Badreddine.Assouar@univ-lorraine.fr, Tel: +33 3 83 68 49 05*

In the supplementary material, the detailed procedures to analytically derive the wave dispersion equations from the equivalent mass-spring system shown in the manuscript is provided. will be given. Also, the issue of the stability of the proposed metamaterial is discussed. The transition from the positive group velocity to the negative group velocity by adjusting the metamaterial's length is also shown.

Analytic investigation of the general mass-spring system for flexural waves

First, the general mass-spring system shown in Fig. 1 (b) is considered to analytically evaluate the general wave dispersion equation of flexural waves. For the convenience, Fig. 1 (b) is re-plotted with detailed motions in Fig. S1. From the vertical and rotational motions of the mass in the nth unit cell shown in Fig. S1, the following equations of motions can be derived;

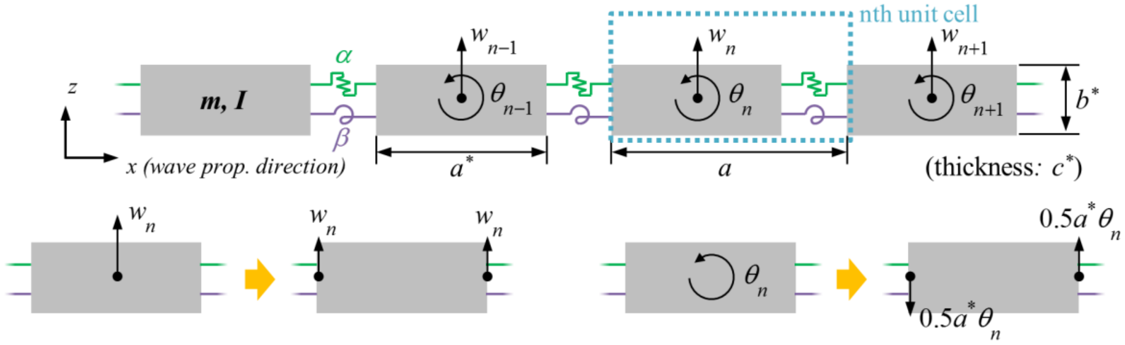

Fig. S1. A periodic mass-spring system for the general flexural wave mode.

$$m\frac{\partial^2 w_n}{\partial t^2}=\alpha(w_{n+1}-0.5a^*\theta_{n+1}-u_n-0.5a^*\theta_n)+\alpha(w_{n-1}+0.5a^*\theta_{n-1}-u_n+0.5a^*\theta_n), \quad \text{(S1)}$$

$$\begin{aligned}I\frac{\partial^2 \theta_n}{\partial t^2}=&\beta(\theta_{n+1}-\theta_n)+\beta(\theta_{n-1}-\theta_n)+0.5a^*\alpha(w_{n+1}-0.5a^*\theta_{n+1}-u_n-0.5a^*\theta_n)\\&-0.5a^*\alpha(w_{n-1}+0.5a^*\theta_{n-1}-u_n+0.5a^*\theta_n)\end{aligned}. \quad \text{(S2)}$$

Here, $m=\rho a^*b^*c^*$ and $I=\rho a^*b^*c^*(a^{*2}+b^{*2})/12$ are the mass and the rotational inertia while ρ is the density[S1]. Also, w_n and θ_n are the *z*-directional displacement and the rotation of the nth unit cell, respectively, and *t* denotes time. Under the time harmonic wave motion with an angular frequency ω, one can assume the periodic condition as $w_{n+1}=\exp(-ika)w_n$ and $\theta_{n+1}=\exp(-ika)\theta_n$, where k is the wavenumber[S2]. Substituting the periodic condition to equations (S1, 2) yields

$$-m\omega^2 w_n=\alpha(\exp(ika)+\exp(-ika)-2)w_n+0.5a^*\alpha(\exp(ika)-\exp(-ika))\theta_n, \quad \text{(S3)}$$

$$\begin{aligned}-I\omega^2\theta_n=&\beta(\exp(ika)+\exp(-ika)-2)\theta_n+0.5a^*\alpha(\exp(-ika)-\exp(ika))w_n\\&-(0.5a^*)^2\alpha(\exp(ika)+\exp(-ika)+2)\theta_n\end{aligned}, \quad \text{(S4)}$$

which is same as equations (1-4).

As in the manuscript, equations (S3, 4) can be more simplified by assuming $a^*=a<<1$, i.e., $\exp(ika)+\exp(-ika)\sim 2-(ka)^2$ and $\exp(ika)-\exp(-ika)\sim 2ika$. Also, with the assumption of $a^*=a<<1$, the mass m and the rotational inertia I can be replaced as

$$m = \rho a^* b^* c^* \sim \rho a b^* c^* = \rho a A_b , \quad \text{(S5)}$$

$$I = \rho a^* b^* c^* (a^{*2} + b^{*2}) / 12 \sim \rho a b^{*3} c^* / 12 = \rho a I_b , \quad \text{(S6)}$$

where $A_b = b^* c^*$ and $I_b = b^{*3} c^* / 12$ are the cross-sectional area and the bending momentum of inertia of the beam structure, respectively. With these assumptions, one can derive the following equations from equations (S3, 4) as

$$-\rho A_b \omega^2 w_n = -\alpha a k^2 w_n + i\alpha k a \theta_n , \quad \text{(S7)}$$

$$\begin{aligned} -\rho I_b \omega^2 \theta_n &= -\beta a k^2 \theta_n - i\alpha a k w_n - \alpha a \theta_n + 0.25 \alpha a^3 k^2 \theta_n \\ &\sim -\beta a k^2 \theta_n - i\alpha a k w_n - \alpha a \theta_n \end{aligned} . \quad \text{(S8)}$$

In equation (S8), the high order term of a^3 is assumed to be zero since $a << 1$. As a result, equation (5) in the manuscript can be obtained as

$$\begin{bmatrix} \alpha a k^2 - \rho \omega^2 A_b & -i\alpha k a \\ i\alpha k a & \beta a k^2 + \alpha a - \rho \omega^2 I_b \end{bmatrix} \begin{bmatrix} w_n \\ \theta_n \end{bmatrix} = 0 . \quad \text{(S9)}$$

The wave dispersion equation can be obtained by setting the determinant of the matrix in equation (S9) to be zero, as shown in equation (5).

In fact, equation (S9) is exactly the same as the wave dispersion equation of the Timoshenko beam theory[S3]. To clearly show this point, it should be noted that for the homogeneous continuum beam structure, the spring coefficients α and β are defined as[S1]

$$\alpha = G A_b \kappa / a , \quad \beta = E I_b / a \quad \text{(S10)}$$

where G and E are the shear modulus and the Young's modulus of the beam, respectively, and κ is the shear correction factor that is defined from the beam's cross-sectional geometry. Substituting equation (S10) to equation (S9) yields

$$(G A_b \kappa k^2 - \rho A_b \omega^2) w_n - i G A_b \kappa k \theta_n = 0 , \quad \text{(S11)}$$

$$i G A_b \kappa k w_n + (E I_b k^2 + G A_b \kappa - \rho I_b \omega^2) \theta_n = 0 , \quad \text{(S12)}$$

which is exactly identical to the wave dispersion relation from the Timoshenko beam theory. This equivalence also validates equation (S9) used in this work.

Analytic investigation of the mass-spring system of the proposed metamaterial

Now, let's focus on the equivalent mass-spring system of the proposed metamaterial shown in Fig. 3 (b). Fig. S2 shows the equivalent mass-spring system shown in Fig. 3 (b) with the detailed motions of the blocks. For the convenience, the points at which the spring is attached on the block are named as the point 1, 2, 3 and 4, as in Fig. S2. Here, the spring γ has the inclination of Φ with respect to the x axis. From the well-known coordinate transformation, the force-displacement relation of the spring γ attached on the point 1 and 4 can be written in the x-z axis as

$$\begin{bmatrix} F_x \\ F_z \end{bmatrix} = \gamma \begin{bmatrix} \cos^2\Phi & \cos\Phi\sin\Phi \\ \cos\Phi\sin\Phi & \sin^2\Phi \end{bmatrix} \begin{bmatrix} u \\ w \end{bmatrix}, \tag{S13}$$

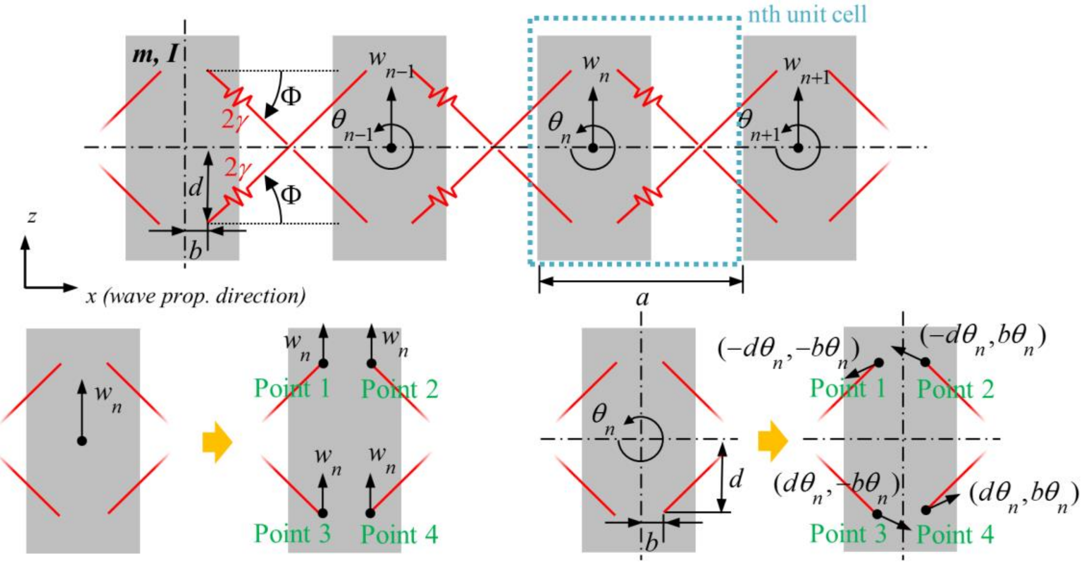

Fig. S2. The equivalent mass-spring system of the proposed hinge connection based metamaterial under the flexural elastic wave propagating along the x axis.

and in the same manner, the force-displacement relation of the spring attached on the point 2 and 3 is

$$\begin{bmatrix} F_x \\ F_z \end{bmatrix} = \gamma \begin{bmatrix} \cos^2\Phi & -\cos\Phi\sin\Phi \\ -\cos\Phi\sin\Phi & \sin^2\Phi \end{bmatrix} \begin{bmatrix} u \\ w \end{bmatrix}. \tag{S14}$$

Based on equations (S13, 14), the *x*- and *z*- directional forces, F_x and F_z, applied on the nth unit cell can be derived as followings;

- At point 1,

$$F_x^1 = \gamma\cos^2\Phi(d\theta_{n-1} + d\theta_n) + \gamma\cos\Phi\sin\Phi(w_{n-1} + b\theta_{n-1} - w_n + b\theta_n), \tag{S15}$$

$$F_z^1 = \gamma\cos\Phi\sin\Phi(d\theta_{n-1} + d\theta_n) + \gamma\sin^2\Phi(w_{n-1} + b\theta_{n-1} - w_n + b\theta_n), \tag{S16}$$

- At point 2,

$$F_x^2 = \gamma\cos^2\Phi(d\theta_{n+1} + d\theta_n) - \gamma\cos\Phi\sin\Phi(w_{n+1} - b\theta_{n+1} - w_n - b\theta_n), \tag{S17}$$

$$F_z^2 = -\gamma\cos\Phi\sin\Phi(d\theta_{n-1} + d\theta_n) + \gamma\sin^2\Phi(w_{n+1} - b\theta_{n+1} - w_n - b\theta_n), \tag{S18}$$

- At point 3,

$$F_x^3 = \gamma\cos^2\Phi(-d\theta_{n-1} - d\theta_n) - \gamma\cos\Phi\sin\Phi(w_{n-1} + b\theta_{n-1} - w_n + b\theta_n) = -F_x^1, \tag{S19}$$

$$F_z^3 = -\gamma\cos\Phi\sin\Phi(-d\theta_{n-1} - d\theta_n) + \gamma\sin^2\Phi(w_{n-1} + b\theta_{n-1} - w_n + b\theta_n) = F_z^1, \tag{S20}$$

- At point 4,

$$F_x^4 = \gamma\cos^2\Phi(-d\theta_{n+1} - d\theta_n) + \gamma\cos\Phi\sin\Phi(w_{n+1} - b\theta_{n+1} - w_n - b\theta_n) = -F_x^2, \tag{S21}$$

$$F_z^4 = \gamma\cos\Phi\sin\Phi(-d\theta_{n+1} - d\theta_n) + \gamma\sin^2\Phi(w_{n+1} - b\theta_{n+1} - w_n - b\theta_n) = F_z^2, \tag{S22}$$

Note that due to the hinge connection, no moment is induced at each point. With the forces exerted on each point, the total forces and moment of the block in the nth unit cell, F_x^{tot}, F_z^{tot} and M_y^{tot}, can be derived as

$$F_x^{tot} = F_x^1 + F_x^2 + F_x^3 + F_x^4 = F_x^1 + F_x^2 - F_x^1 - F_x^2 = 0, \tag{S23}$$

$$\begin{aligned} F_z^{tot} &= F_z^1 + F_z^2 + F_z^3 + F_z^4 = 2F_z^1 + 2F_z^2 \\ &= 2\gamma\cos\Phi\sin\Phi(d\theta_{n-1} - d\theta_{n+1}) + 2\gamma\sin^2\Phi(w_{n+1} + w_{n-1} - b\theta_{n+1} + b\theta_{n-1} - 2w_n) \end{aligned}, \tag{S24}$$

$$\begin{aligned} M_y^{tot} &= -dF_x^1 - dF_x^2 + dF_x^3 + dF_x^4 - bF_z^1 + bF_z^2 - bF_z^3 + bF_z^4 \\ &= 2\gamma\sin\Phi(b\sin\Phi + d\cos\Phi)(w_{n+1} - w_{n-1}) \\ &\quad - 2\gamma(d^2\cos^2\Phi + 2bd\cos\Phi\sin\Phi + b^2\sin^2\Phi)(\theta_{n+1} + \theta_{n-1} + 2\theta_n) \end{aligned}. \tag{S25}$$

Assuming the periodic condition as $w_{n+1} = \exp(-ika)w_n$ and $\theta_{n+1} = \exp(-ika)\theta_n$, the following wave dispersion equation, same as equations (8-11), can be obtained;

$$\begin{aligned} -m\omega^2 w_n &= 2\gamma\sin^2\Phi(\exp(-ika) + \exp(ika) - 2)w_n \\ &\quad + 2\gamma\sin\Phi(b\sin\Phi + d\cos\Phi)(\exp(ika) - \exp(-ika))\theta_n \end{aligned}, \tag{S26}$$

$$\begin{aligned} -I\omega^2\theta_n &= 2\gamma\sin\Phi(b\sin\Phi + d\cos\Phi)(\exp(-ika) - \exp(ika))w_n \\ &\quad - 2\gamma(b\sin\Phi + d\cos\Phi)^2(\exp(-ika) + \exp(ika) + 2)\theta_n \end{aligned}. \tag{S27}$$

Stability issue of the proposed metamaterial

As commented in the manuscript, the metamaterial considered in this work should have an instability at zero frequency. To clearly show this point, consider the single unit cell structure connected to the fixed block, as shown in Fig. S3. Here, assume that an arbitrary force or moment is induced at the center of the block. At zero frequency, the force and moment equilibrium equations are written as

$$\begin{bmatrix} F \\ M \end{bmatrix} = \begin{bmatrix} 2\gamma\sin^2\Phi & -2\gamma\sin\Phi(d\cos\Phi + b\sin\Phi) \\ -2\gamma\sin\Phi(d\cos\Phi + b\sin\Phi) & 2\gamma(d\cos\Phi + b\sin\Phi)^2 \end{bmatrix} \begin{bmatrix} w \\ \theta \end{bmatrix}. \tag{S28}$$

However, one can immediately find that this equation does not have any unique solutions since the determinant of the stiffness matrix is zero. The structure can have infinite values of the displacement w and the rotations θ at the zero frequency. Thus, if the proposed structure is

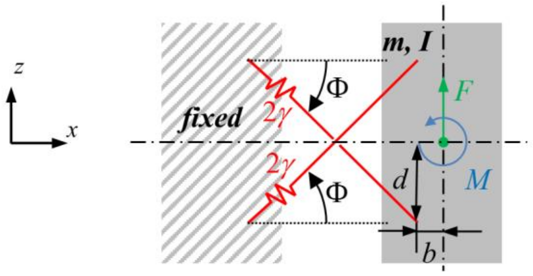

Fig. S3. The single mass-spring system attached to the fixed block.

perfectly fabricated to exhibit the zero rotational stiffness, the structure has an instability at the static limit and cannot sustain its own weight.

However, in reality, the proposed metamaterial can be stable due to the friction. In fact, it is impossible to realize the purely zero rotational stiffness. In the numerical investigations shown in the manuscript, we assumed that there is no friction at the hinge connection so that each block and link can freely rotate. However, in real structure, there exists friction at the hinge connection and moments can be slightly transferred due to the friction, i.e., the structure should have at least small rotational stiffness. Thus, in reality, the proposed metamaterial should have the 'near-zero rotational stiffness', not the 'zero rotational stiffness'. Although it may be small value compared to the other stiffness values, this small rotational stiffness can make the metamaterial stable even at the zero frequency.

In this point, one can have the following question – can we use the findings from the 'zero rotational stiffness' to the actual 'near-zero rotational stiffness' metamaterial? To answer this point, we plot the wave dispersion curves corresponding to β=0, 0.01 and 0.1 in Fig. S4, while α, m and I are all 1. As can be seen here, despite of the nonzero rotational stiffness, the second wave dispersion branch is almost unaltered and still extremely low frequency stop band can be obtained. Although the stop band may not start from the zero frequency, it can be seen that the proposed idea can realize the metamaterial with extremely low stop band.

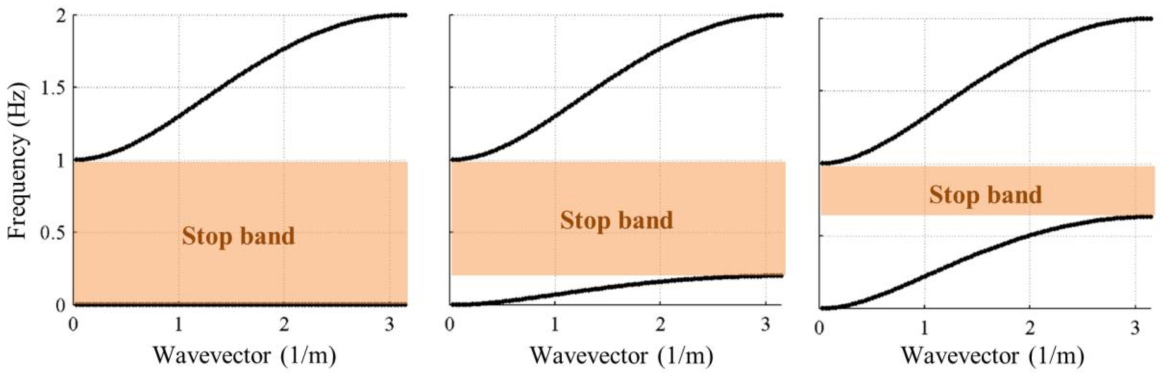

Fig. S4. The wave dispersion curve with β=0 (left), 0.01 (middle) and 0.1 (right).

Detailed geometric parameter of the proposed metamaterials

The detailed geometrical parameters of the proposed metamaterial are shown in Fig. S5 (a). The length of the block l is considered to be 35 mm for the metamaterial having the positive group velocity (those considered in Figs. 4-6) while it is considered to be 75 mm for the metamaterial having the negative group velocity (those considered in Fig. 7, 8).

Based on these parameters, the constants in equation (8) can be defined. In evaluating m and I, the holes inside the block are ignored since their volumes are very small compared to the volume of the block. Thus, $m = 2700\times 0.03\times 0.08\times l$ and $I = m\times(l^2+0.08^2)/12$ are used. Also, the spring coefficient γ is defined from the static finite element analysis shown in Fig. S5 (b). Here, the fixed boundary condition is imposed in the left hole, and a unit force is imposed at the center of the right hole. (Note that due to the hinge connection condition imposed at the right hole, the force exerted at the center of the right hole is equivalently distributed to the boundary of the hole). After that, the displacement of the link structure is measured to calculate the spring coefficient. The resulting constants are summarized as

$$b = (l-0.02)/2 \text{ [m]},\quad d = 0.03 \text{ [m]},\quad \gamma = 1.9216\text{e}5 \text{ [N/m]}, \qquad \text{(S29-31)}$$

$$m = 6.48l \text{ [kg]},\quad I = 0.54l(l^2+6.4e-3) \text{ [kg}\cdot\text{m}^2\text{]},\quad \sin\Phi = 12/13,\quad \cos\Phi = 5/13 \quad \text{(S32-35)}$$

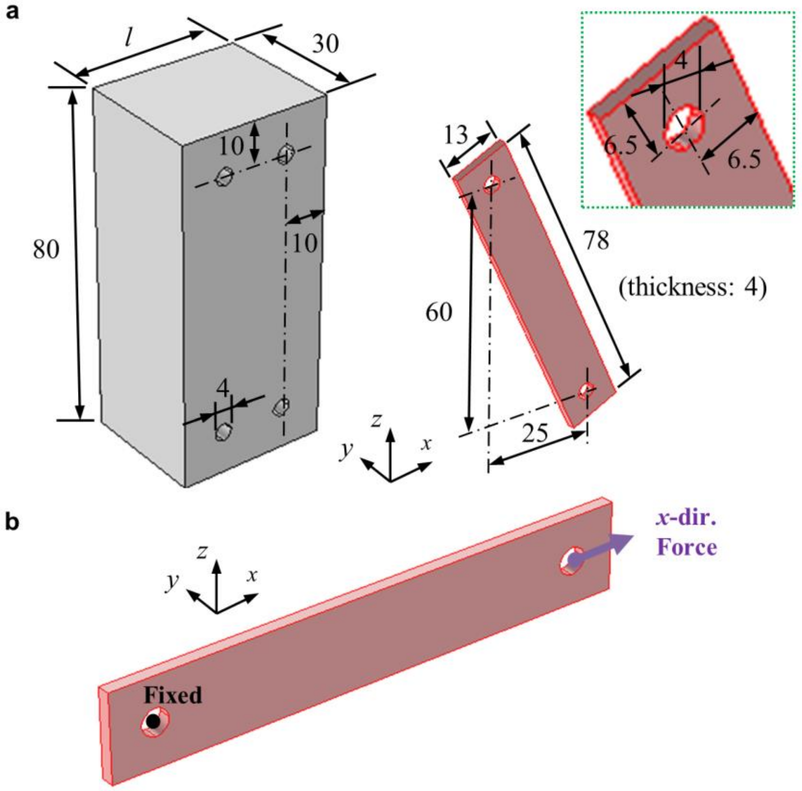

Fig. S5. (a) The detailed geometric parameter of the continuum unit cell structure (the parameters are expressed in mm), (b) the schematic figure of the finite element analysis to evaluate the spring coefficient γ.

Transition between the positive to negative group velocity

In the manuscript, the negative group velocity is achieved by enlarging the length of the blocks (*l* in Fig. S5). Here, we will show the wave dispersion curves of various metamaterials by gradually enlarge the length of the block (*l* in Fig. S5) to see the transition from the positive to the negative group velocity. Fig. S5 shows the wave dispersion curves of the various metamaterials. As can be seen here, one can further design the metamaterial to have the proper positive or negative group velocities, with the quasi-static stop band.

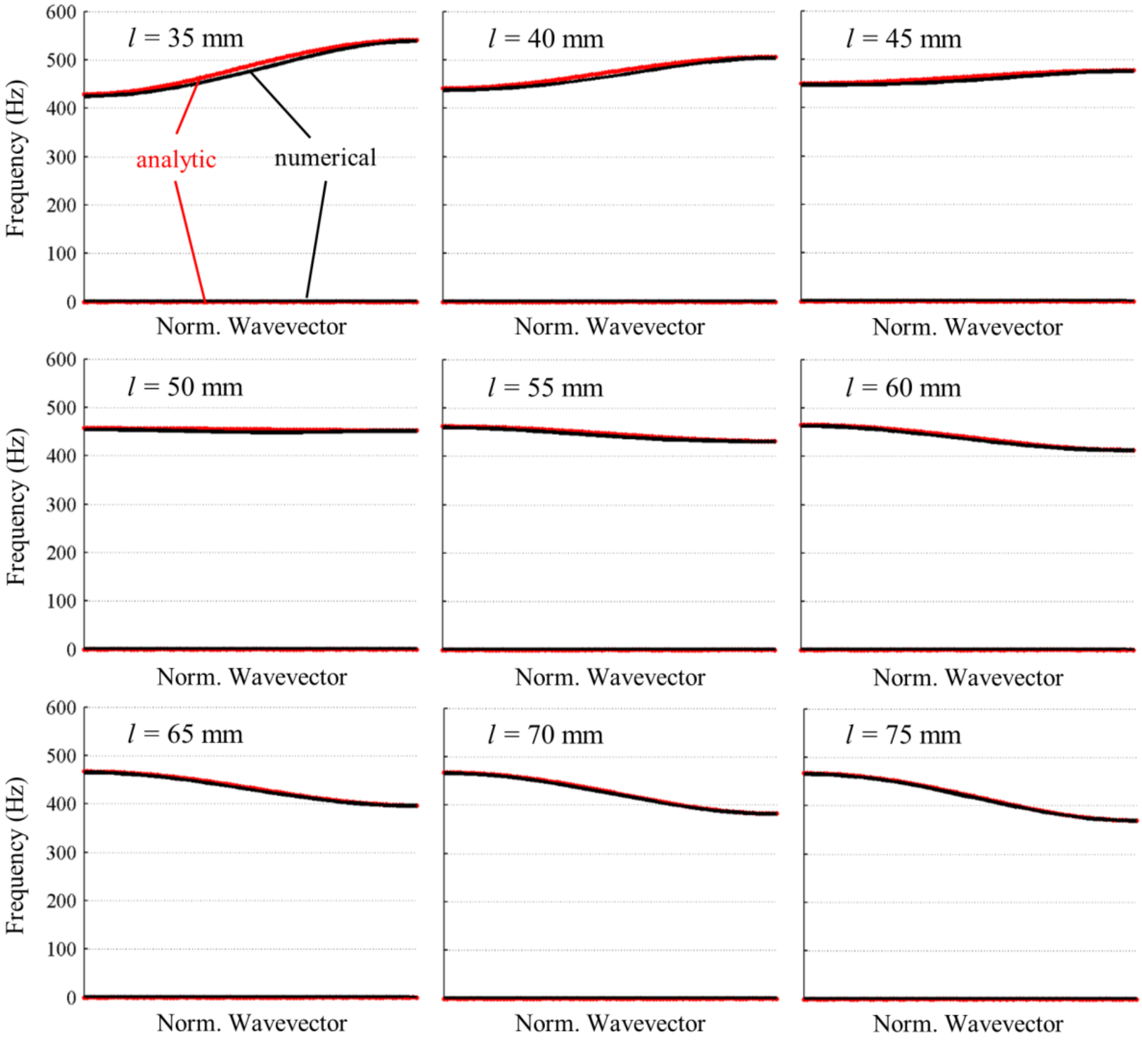

Fig. S6. Plots of the wave dispersion curves calculated with the metamaterials having various length of the block, *l*.

References

S1 Meirovitch, M. *Fundamentals of vibrations*. (McGraw-Hill Companis, Inc., 2001).

S2 Brillouin, L. *Wave propagation in periodic structures*. (Dover Publications, Inc., 1953).

S3 Graff, K. F. *Wave Motion in Elastic Solids* (Dover, New York, 1991).
